# Efficacy and Limitations of Flow Cytometry for the Rapid Diagnosis of Primary Central Nervous System Lymphoma

**DOI:** 10.3390/cancers17223646

**Published:** 2025-11-13

**Authors:** Hikaru Nakamura, Takeshi Hiu, Takeharu Kato, Nozomi Ueki, Ayaka Matsuo, Michiharu Yoshida, Shiro Baba, Kenta Ujifuku, Koichi Yoshida, Hirofumi Koike, Yukishige Hayashi, Hiroo Hasegawa, Koji Ando, Katsunori Yanagihara, Masahiro Nakashima, Yasushi Miyazaki, Takayuki Matsuo

**Affiliations:** 1 Department of Neurosurgery, Nagasaki University Graduate School of Biomedical Sciences, 1-7-1 Sakamoto, Nagasaki 852-8501, Japan; hikaru.nakamura560@gmail.com (H.N.); heygoo25@live.jp (A.M.); michi511leo@yahoo.co.jp (M.Y.); bb46.vader@gmail.com (S.B.); kentaujifuku@hotmail.com (K.U.); kou-yoshida@nagasaki-u.ac.jp (K.Y.); takayuki@nagasaki-u.ac.jp (T.M.); 2 Department of Neurosurgery, Nagasaki Prefecture Shimabara Hospital, 7895 Shimokawashiri, Nagasaki 855-0861, Japan; yukisige1983@icloud.com; 3 Department of Hematology, Nagasaki University Hospital, 1-7-1 Sakamoto, Nagasaki 852-8501, Japan; tkatou@nagasaki-u.ac.jp (T.K.); y-miyaza@nagasaki-u.ac.jp (Y.M.); 4 Department of Tumor and Diagnostic Pathology, Atomic Bomb Disease Institute, Nagasaki University, 1-12-4 Sakamoto, Nagasaki 852-8523, Japan; nozomiueki@nagasaki-u.ac.jp (N.U.); moemoe@nagasaki-u.ac.jp (M.N.); 5 Department of Radiology, Nagasaki University Graduate School of Biomedical Sciences, 1-7-1 Sakamoto, Nagasaki 852-8501, Japan; hkoike@nagasaki-u.ac.jp; 6 Department of Laboratory Medicine, Nagasaki University, Nagasaki 852-8523, Japan; hhase@nagasaki-u.ac.jp (H.H.); k-yanagi@nagasaki-u.ac.jp (K.Y.); 7 Department of Hematology, Unit for Radiation Effects on Humans, Atomic Bomb Disease Institute, Nagasaki University, 1-12-4 Sakamoto, Nagasaki 852-8523, Japan; k-ando@nagasaki-u.ac.jp

**Keywords:** flow cytometry, primary central nervous system lymphoma, diffuse large B-cell lymphoma

## Abstract

Background/Objectives: Primary central nervous system lymphoma (PCNSL) has a markedly high proliferation rate, making early diagnosis and prompt therapeutic intervention essential. To accelerate diagnosis, our institution adopted flow cytometry (FCM) in conjunction with conventional histopathology, and this study therefore evaluated the diagnostic performance of FCM for PCNSL. Methods: We retrospectively analyzed 67 consecutive patients with suspected PCNSL who underwent intraoperative FCM between 2010 and 2023 based on preoperative imaging. B-cell clonality was defined as ≥20% CD19/CD20-positive cells with a κ/λ ratio of >3.0 or <0.5. Results: Using histopathology, we confirmed the presence of PCNSL in 42 patients, all diagnosed as having diffuse large B-cell lymphoma. Six cases (14.3%) were discordant (FCM-D). The sensitivity, specificity, and positive predictive value of FCM were 85.7%, 100%, and 100%, respectively. T-cell markers were significantly elevated in FCM-D cases (*p* < 0.01), although these were pathologically diagnosed as diffuse large B-cell lymphoma based on histology and immunohistochemistry. Conclusions: FCM yields reliable diagnostic information within hours of tissue collection and supports early therapeutic decisions in PCNSL. Discordant results may reflect reactive T-cell infiltration. This is the first study to present detailed subset analyses in PCNSL using FCM in correlation with pathology, underscoring its utility as a rapid diagnostic tool.

## Simple Summary

Primary central nervous system lymphoma is a form of aggressive brain tumor that requires rapid diagnosis to enable timely treatment. Although accurate, the results of conventional histopathology often take several days. In this study, we evaluated the usefulness of flow cytometry, which can deliver diagnostic information within hours. We retrospectively analyzed 67 patients with suspected primary central nervous system lymphoma and compared the flow cytometry findings with the final pathology; most cases confirmed by pathology were correctly identified by flow cytometry, which showed its high sensitivity and perfect specificity, although some cases were discordant due to reactive T-cell infiltration. Importantly, this is the first study to provide detailed subset analyses of lymphocytes in primary central nervous system lymphoma using flow cytometry. These results demonstrate that flow cytometry is a valuable complement to standard pathology, offering rapid and reliable diagnostic guidance that can accelerate treatment decisions and improve patient care.

## 1. Introduction

Primary central nervous system lymphoma (PCNSL) is a rare extranodal non-Hodgkin lymphoma, accounting for approximately 4% of primary brain tumors and 1% of all non-Hodgkin lymphomas. Most PCNSL cases are diffuse large B-cell lymphomas (DLBCLs), characterized by rapid proliferation and an aggressive clinical course [1,2,3,4,5]. PCNSL incidence has risen, particularly among immunocompetent older adults, reflecting demographic shifts and advances in diagnostic imaging [1,6].

Clinically, PCNSL manifests with diverse, nonspecific neurological symptoms—such as cognitive decline, hemiparesis, seizures, and visual disturbances—that may resemble glioblastoma, demyelinating disease, or metastases. Early and accurate diagnosis is crucial as PCNSL responds to chemotherapy and radiotherapy, especially high-dose methotrexate (HD-MTX)-based regimens [1,2,6].

Patients who receive prompt therapy demonstrate improved progression-free and overall survival, whereas diagnostic delays correlate with poorer outcomes [1].

There are various reports addressing state-of-the-art imaging-based approaches; however, diagnosis remains challenging [7,8,9,10], as there are no disease-specific signs. MRI typically reveals homogeneously enhanced deep-seated lesions, but lacks specificity [1,6,11]. PET/CT can exclude systemic lymphoma but is insufficient for intracranial diagnosis [12]. CSF cytology offers limited sensitivity and molecular approaches such as MYD88 mutation detection or interleukin-10 and C-X-C motif chemokine ligand 13 (CXCL13) quantification are promising but not widely adopted in clinical practice [13,14,15,16]. Consequently, stereotactic biopsy remains the diagnostic gold standard. However, histopathology requires fixation, embedding, staining, and immunohistochemistry, delaying treatment initiation by several days [17].

Established in hematology for diagnosing systemic lymphomas, flow cytometry (FCM) provides rapid immunophenotyping. By assessing surface markers and κ/λ ratios, it can determine B-cell clonality within hours of tissue collection [18]. Several reports have explored intraoperative FCM for CNS lymphomas, indicating that it can differentiate PCNSLs from other tumors and reduce diagnostic delays [19,20,21,22].

Nonetheless, discordant cases occur, often due to reactive T-cell infiltration, including reactive perivascular T-cell infiltration (RPVI)—a histological hallmark of PCNSLs—that may obscure B-cell clonality and yield false negatives [19,23,24].

To address these limitations, we retrospectively analyzed patients who underwent intraoperative FCM for suspected PCNSL at our institution. We aimed to evaluate diagnostic accuracy, characterize discordant cases, and integrate immunophenotypic subset analyses with histopathology. To our knowledge, this is the first study to correlate FCM with pathology in PCNSL at the subset level, thereby clarifying the immunological basis of diagnostic discordance.

## 2. Materials and Methods

### 2.1. Study Design and Patients

For this retrospective cohort study, we included consecutive patients who underwent surgical biopsy or resection for suspected PCNSL at Nagasaki University Hospital between January 2010 and December 2023. Inclusion criteria were (1) preoperative neuroimaging (MRI or CT) suggestive of PCNSL, characterized by homogeneous gadolinium enhancement, deep-seated lesion distribution, or other typical radiological features, and (2) the availability of intraoperative specimens to process for FCM in addition to routine histopathology. Patients with systemic lymphoma, secondary CNS involvement, or insufficient tumor tissue were excluded.

### 2.2. Surgical Procedures and Tissue Handling

All patients underwent either stereotactic biopsy or open resection, depending on lesion characteristics and clinical condition. Tumor specimens were divided: one portion was processed for histopathology and the other was immediately prepared for FCM. For the latter, tissue was mechanically dissociated in RPMI-1640 medium supplemented with 10% fetal bovine serum, filtered through a 40 μm mesh, and processed within one hour after collection to preserve antigenicity.

### 2.3. Flow Cytometry Protocol

Cell suspensions were stained with a panel of monoclonal antibodies targeting B- and T-cell surface markers, including CD3 (clone SK7, number 347347), CD4 (RPA-T4, 3338054), CD5 (UCHT2, 340696), CD8 (Leu-2a, 340046), CD10 (HI10a, 340921), CD19 (4G7, 347543), and CD20 (L27, 346595) (BD Biosciences, San Jose, CA, USA). Light chain restriction was assessed using antibodies against κ and λ immunoglobulin light chains (F0434 and F0435) (Agilent Technologies, Inc., Santa Clara, CA, USA). Data acquisition was performed using an FACSLyric Flow Cytometer (BD Biosciences), and analysis was carried out with FACSuit software (Version 1.2.1) (BD Biosciences).

Consistent with established criteria, B-cell clonality was defined as ≥20% of lymphocytes expressing CD19 or CD20, combined with an abnormal κ/λ ratio (>3.0 or <0.5) [19,21]. T-cell markers were analyzed to evaluate reactive perivascular infiltration. Discordance (FCM-D) was defined as when FCM failed to demonstrate B-cell clonality despite histopathological confirmation of PCNSL.

### 2.4. Histopathological Examination

Specimens were fixed in 10% neutral buffered formalin, paraffin-embedded, and stained with hematoxylin and eosin (HE). For immunohistochemistry (IHC), we employed antibodies against CD3, CD10, CD20, BCL-6, MUM1, and Ki-67, following standard protocols. Diagnoses were established by board-certified neuropathologists in accordance with the World Health Organization (WHO) classification of hematopoietic and lymphoid tumors [25]. Cases showing reactive perivascular T-cell infiltration (RPVI) were specifically recorded.

### 2.5. Clinical and Radiological Data

Baseline demographic and clinical data included age, sex, Karnofsky Performance Status (KPS), biopsy type, and tumor number (single vs. multiple). MRI findings, including contrast enhancement patterns, hemorrhage, and calcification, were independently reviewed by two neuroradiologists.

### 2.6. Statistical Analysis

Continuous variables were tested for normality using the Shapiro–Wilk test. As most variables were not normally distributed, they were expressed as the median and compared using the Mann–Whitney U test. Categorical variables were evaluated using Fisher’s exact or Chi-square tests, as appropriate.

The diagnostic accuracy of FCM was calculated using histopathology as the reference standard, with sensitivity, specificity, positive predictive value, and negative predictive value reported. Subgroup analyses compared FCM-concordant (FCM-C) and FCM-discordant (FCM-D) patients regarding their immunophenotypic and clinical features. Statistical analyses were performed with EZR (Saitama Medical Center, Jichi Medical University, Saitama, Japan), a modified version of R Commander for biostatistics [26], with statistical significance set at *p* < 0.05.

### 2.7. Ethical Considerations

This study was approved by the Institutional Review Board of Nagasaki University Hospital (Approval No. 2106117). Owing to its retrospective design and use of anonymized data, informed consent was waived. All procedures adhered to the principles of the Declaration of Helsinki.

## 3. Results

### 3.1. Patient Characteristics

**Figure 1:**
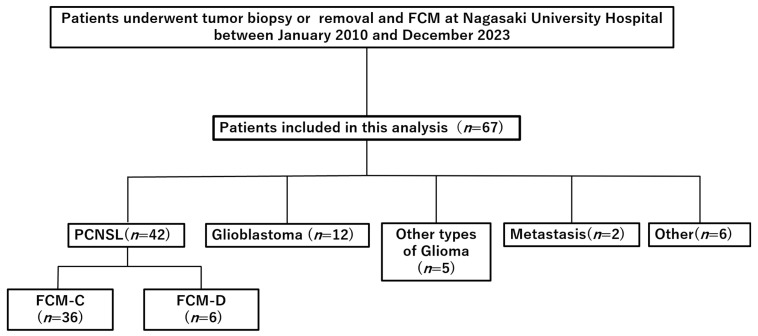
Flow diagram of patient selection. Retrospective cohort of patients who underwent tumor biopsy or removal with intraoperative flow cytometry (FCM) at Nagasaki University Hospital between January 2010 and December 2023. Abbreviations: FCM, flow cytometry; PCNSL, primary central nervous system lymphoma; FCM-C, FCM concordant; FCM-D, FCM discordant.

Between January 2010 and December 2023, 67 patients with suspected PCNSL underwent biopsy or tumor removal with intraoperative FCM. Histopathological evaluation confirmed PCNSL in 42 patients, while 25 were diagnosed with other conditions: glioblastoma (*n* = 12), other gliomas (*n* = 5), metastases (*n* = 2), and miscellaneous tumors, including meningioma or inflammatory lesions (*n* = 6) (Figure 1). All confirmed PCNSL cases were classified as DLBCL.

Among the 42 patients with PCNSL, FCM and histopathology were concordant in 36 (85.7%, FCM-C) and discordant in 6 (14.3%, FCM-D). Baseline demographic and clinical data are presented in Table 1. The median age of the PCNSL cohort was 71.5 years, with no significant difference between FCM-C (72.0 years) and FCM-D (66.5 years) groups (*p* = 0.35). Male patients accounted for 58.3% of FCM-C and 50.0% of FCM-D cases (*p* = 1.0). Most diagnoses were obtained via stereotactic needle biopsy (92.9%), and the median of preoperative Karnofsky Performance Status (KPS) was 50 overall. Radiological findings included homogeneous gadolinium enhancement in 69.0% of PCNSL cases, hemorrhage in 40.5%, and no calcifications. None of these features differed significantly between FCM-C and FCM-D groups.

### 3.2. Diagnostic Accuracy of Flow Cytometry

We evaluated the diagnostic performance of FCM using histopathology as the reference standard. Of the 42 PCNSL cases, 36 were FCM-C, while all 25 non-PCNSL tumors were FCM-D. Sensitivity was 85.7% (36/42), specificity 100% (25/25), positive predictive value 100% (36/36), and negative predictive value 80.6% (25/31). These results underscore the high specificity of FCM, as no false positives occurred; however, false negatives were observed in 14.3% of patients with PCNSL, highlighting the need to interpret discordant results cautiously.

### 3.3. Lymphocyte Subset Analysis

**Figure 2:**
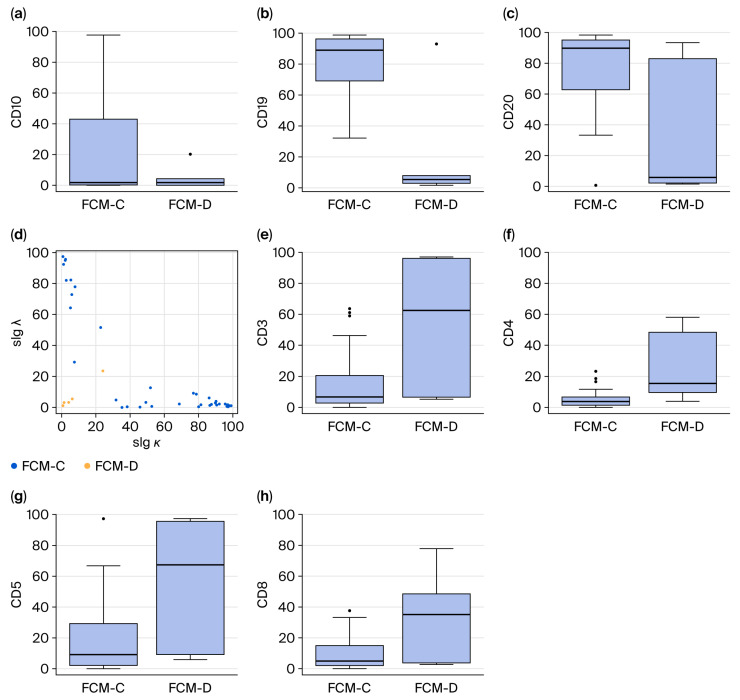
Lymphocyte subset analysis (**a**–**c**,**e**–**h**) and scatter plots (**d**) determined by FCM. Percentages of B-cell surface markers (**a**–**c**). Scatter plot of slgκ and slgλ (**d**). Percentages of T-cell surface markers (**e**–**h**). Abbreviations: FCM-C, flow cytometry concordant; FCM-D, flow cytometry discordant; slgκ, surface immunoglobulin κ; slgλ, surface immunoglobulin λ.

Flow cytometric analysis revealed clear differences between FCM-C and FCM-D (Figure 2). In FCM-C, B-cell markers were consistently elevated: CD10 positivity averaged 23.8 ± 35.3% versus 4.8 ± 7.7% in FCM-D (*p* < 0.01); CD19 was 81.1 ± 19.6% in FCM-C compared with 19.4 ± 36.1% in FCM-D (*p* < 0.01); and CD20 was 76.8 ± 24.2% in FCM-C versus 32.0 ± 43.8% in FCM-D (*p* < 0.01). These findings demonstrate strong B-cell clonality, combined with abnormalities in the k/λ ratio in FCM-C.

Conversely, T-cell markers predominated in FCM-D: CD3 expression averaged 55.1 ± 41.0% in FCM-D versus 15.4 ± 18.2% in FCM-C (*p* < 0.01). CD4 also increased (25.3 ± 22.5% vs. 5.3 ± 5.4%, *p* < 0.01), as did CD5 (57.1 ± 40.6% vs. 17.3 ± 22.4%, *p* < 0.01) and CD8 (33.9 ± 28.6% vs. 8.7 ± 9.7%, *p* < 0.01). These results suggest that T-cell predominance in discordant cases is obscured B-cell clonality in FCM.

### 3.4. Histopathological Correlates of Discordant Cases

**Figure 3:**
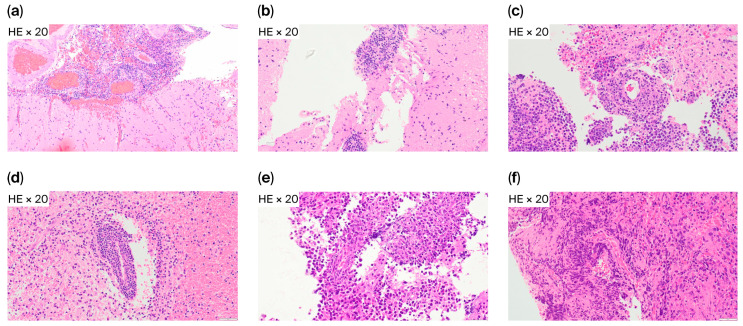
Reactive perivascular T-cell infiltration in FCM-D cases. Representative HE staining from six FCM-D cases. Reactive perivascular T-cell infiltration (RPVI) was present in four cases (**a**–**d**) and absent in two (**e**,**f**). Abbreviations: FCM-D, FCM discordant; HE, hematoxylin and eosin; RPVI, reactive perivascular T-cell infiltration.

Histopathology provided additional insights into the six FCM-D cases. HE staining demonstrated RPVI in four patients (66.7%) (Figure 3), while in the other two cases, tumor tissue showed diffuse lymphomatous infiltration without marked RPVI, indicating that sampling error or low tumor cell content may also explain discordance.

**Figure 4:**
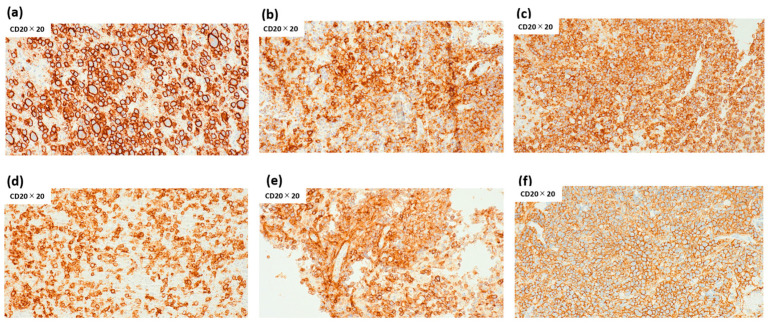
Percentage of intratumoral CD20-positive lymphocytes in FCM-D cases. Percentages of intratumoral CD20-positive lymphocytes were 79% (**a**), 83% (**b**), 93% (**c**), 72% in (**d**), 87% (**e**), and 98% (**f**), with a median of 85% (range 72–98%). Abbreviations: FCM-D, FCM discordant.

Despite the lack of FCM-detected clonality, all six FCM-D cases were confirmed as B-cell lymphoma by IHC. CD20 immunostaining revealed high intratumoral B-cell proportions, with positivity ranging from 72% to 98% (median 85%) (Figure 4). CD3 immunostaining revealed low T-cell proportions, with positivity ranging from 2.6% to 53.4% (median 6.4%), although one case could not be evaluated. These findings confirm that FCM-D cases represented DLBCL.

## 4. Discussion

The results of our study demonstrate that intraoperative FCM is a rapid and highly specific diagnostic tool for PCNSL, with sensitivity of 85.7% and specificity of 100%. These results are consistent with previous investigations of FCM in stereotactic brain biopsies [19,20,21,22,27] but extend prior work by incorporating detailed subset analyses and correlating discordant findings with histopathological features. We identify an important biological factor—immune microenvironmental influence—that may confound cytometric evaluation in PCNSL.

### 4.1. Diagnostic Value of FCM

FCM’s high specificity and positive predictive value underscore it as a strong diagnostic adjunct. The absence of false positives indicates that once B-cell clonality is detected, clinicians can confidently initiate lymphoma-directed therapy. This is particularly valuable in neurosurgical practice, where delays in chemotherapy can worsen outcomes [1]. Although histopathology remains definitive, it requires several days for tissue processing and immunohistochemistry to be conducted. By contrast, FCM yields results within hours, enabling earlier treatment planning [17,19,20,21].

### 4.2. Discordant Cases and the Role of the Immune Microenvironment

A unique contribution of this study is the characterization of discordant cases. Although histopathology confirmed B-cell lymphomas, FCM-D cases demonstrated the predominance of T-cell markers. Histological review revealed RPVI in most discordant cases, supporting the interpretation that immune microenvironmental factors can obscure B-cell clonality. RPVI has been described as characteristic of PCNSL and may carry prognostic relevance [2,23,24,28,29]. Our findings emphasize that reactive T-cell infiltrates can alter cytometric profiles, masking underlying lymphomas.

The immunological implications of RPVI warrant attention. Several reports suggest that perivascular T-cell infiltrates not only confound diagnosis but may also represent an active host immune response with potential survival benefits [2,28,29].

This dual role illustrates the complexity of the PCNSL microenvironment. Clinically, a T-cell-dominant FCM profile should not exclude PCNSL, particularly when radiological and clinical evidence is compelling. Biologically, RPVI may provide insight into immune surveillance mechanisms that could be harnessed therapeutically [2,23,24,28,29].

### 4.3. Comparison with the Previous Literature

The sensitivity and specificity observed in our study are comparable to those reported by Takeuchi et al. [19] and Inoue et al. [21]. Our results align with those of Cordone et al., who demonstrated the feasibility of stereotactic biopsy FCM and showed that it has a high degree of agreement (89%) with immunohistochemistry in brain lymphoma identification [22]. For CNS lymphomas, Koriyama et al. further showed that intraoperative FCM differentiates PCNSL from glioblastoma—a distinction often difficult on imaging [30]. However, these studies did not explore discordance in detail. By integrating subset analysis with histopathological correlation, our study identifies reactive T-cell infiltration as a major contributor to discordance and emphasizes cautious interpretation of FCM-negative results.

### 4.4. Clinical Implications

Incorporating FCM into intraoperative workflows offers several advantages. First, it enables rapid confirmation of B-cell clonality, facilitating early chemotherapy initiation. Second, combining FCM with intraoperative IHC and cytology may enhance accuracy in cases with marked T-cell infiltration [21,22].

Recognizing that discordant FCM results may reflect an immune-rich tumor microenvironment also carries prognostic and counseling-related implications. As the field of oncology moves toward precision strategies, incorporating immunological features such as RPVI into diagnostic and prognostic models may become increasingly relevant [2,23,24,28,29].

The authors of future studies should validate FCM protocols in multicenter prospective cohorts, standardize gating strategies, and establish universally accepted cut-off values. Ultimately, integrating FCM with histopathology, molecular diagnostics, and advanced imaging may provide a multimodal framework for the rapid, reliable diagnosis of PCNSL.

### 4.5. Limitations

This study has limitations inherent to its retrospective, single-center design. The small number of discordant cases (*n* = 6) restricted statistical power for subgroup analyses. Technical variability, including tissue handling and sample size, may also have affected our results. Moreover, our clonality thresholds (≥20% CD19/CD20 with κ/λ > 3.0 or <0.5) were adapted from prior reports and require multicenter validation.

## 5. Conclusions

Flow cytometry is a rapid, accurate, and highly specific adjunct to histopathology for diagnosing PCNSL. Although discordance may arise from reactive T-cell infiltration and there remains room for improvement in this area, incorporating FCM into intraoperative workflows can expedite therapy, improve outcomes, and support multimodal diagnostic strategies.

## Figures and Tables

**Table 1:** Baseline patient characteristics.

	Total (*n* = 42)	FCM-C (*n* = 36)	FCM-D (*n* = 6)	*p*-Value
Age, median (range)	71.5 (51–85)	72.0 (51–85)	66.5 (57–78)	0.35
Male sex, *n* (%)	24 (57.1)	21 (58.3)	3 (50.0)	1
Needle biopsy, *n* (%)	39 (92.9)	34 (94.4)	5 (83.3)	0.38
Preoperative KPS, median (range)	50 (30–80)	50 (30–80)	40 (30–70)	0.18
Tumor number—single, *n* (%)	24 (57.1)	21 (58.3)	3 (50.0)	1
Tumor number—multiple, *n* (%)	18 (42.9)	15 (41.7)	3 (50.0)	1
MRI findings—homogeneous Gd enhancement, *n* (%)	29 (69.0)	25 (69.4)	4 (66.7)	1
MRI findings—hemorrhage, *n* (%)	17 (40.5)	13 (36.1)	4 (66.7)	0.2
MRI findings—calcification, *n* (%)	0 (0)	0 (0)	0 (0)	N/A

Abbreviations: FCM-C, flow cytometry concordant; FCM-D, flow cytometry discordant; KPS, Karnofsky Performance Status; MRI, magnetic resonance imaging; Gd, gadolinium; N/A, not applicable.

## Data Availability

All relevant data are included within the paper.

## Abbreviations

The following abbreviations are used in this manuscript:

PCNSL	Primary Central Nervous System Lymphoma
DLBCL	Diffuse Large B-Cell Lymphoma
HD-MTX	High-Dose Methotrexate
MRI	Magnetic Resonance Imaging
PET/CT	Positron Emission Tomograph/Computed Tomography
CSF	Cerebrospinal Fluid
CXCL13	C-X-C Motif Chemokine Ligand 13
FCM	Flow Cytometry
RPVI	Reactive Perivascular T-Cell Infiltration
FCM-D	Flow Cytometry Discordant
HE	Hematoxylin and Eosin
IHC	Immunohistochemistry
WHO	World Health Organization
KPS	Karnofsky Performance Status
SD	Standard Deviation
